# Exploring prenatal care experiences in Ontario, Canada: An equity-oriented qualitative study

**DOI:** 10.1371/journal.pone.0345200

**Published:** 2026-03-30

**Authors:** Zeenat Ladak, Muzzammil Hooda, Tolulope Ojo, Daphne To, Rochelle Simmons, Jennifer Shuldiner, Ummul-Kiram Patrawala, Emily Nicholas Angl, Celia Laur, Mehdiya Hemani, Olesya Falenchuk, Richard Volpe, Noah M. Ivers

**Affiliations:** 1 University of Toronto, Toronto, Ontario, Canada; 2 Women’s College Hospital Institute for Health System Solutions & Virtual Care, Toronto, Ontario, Canada; 3 Western University, London, Ontario, Canada; 4 The World of my Baby (WOMB) Clinic, Milton, Ontario, Canada; 5 ENA Consulting, Toronto, Ontario, Canada; 6 Department of Obstetrics and Gynaecology, Mackenzie Health Hospitals, Vaughan, Ontario, Canada; 7 Women’s College Hospital, Toronto, Ontario, Canada; Ben-Gurion University of the Negev Faculty of Health Sciences, ISRAEL

## Abstract

**Introduction:**

The extent to which prenatal care is equity-oriented significantly influences patient satisfaction, care-seeking decisions, and health outcomes for both parent and newborn. The social determinants of health shape care experiences, disproportionately affecting marginalized populations (e.g., low-income, rural, racial minorities). This study explores prenatal patients’ care experiences and examines the impact of health inequities on these experiences.

**Methods:**

This patient-partner-oriented, cross-sectional, qualitative study was guided by Cochrane’s PROGRESS-Plus equity framework and carried out using a pragmatist paradigm. Purposeful, maximum variation sampling recruited individuals in Ontario, Canada who had been pregnant or experienced pregnancy loss within the last 12 months. Participants completed a 5-minute sociodemographic survey and a 1-hour semi-structured interview on their prenatal care experiences and equity impacts. Analysis included descriptive statistics, and inductive and deductive content analysis.

**Results:**

This study included 18 participants, half identifying as racial minorities. All participants interacted with a healthcare professional during pregnancy; most were followed by a primary care physician (n = 16), half by an obstetrician (n = 9), half by a nurse (n = 9), and/or eight by a midwife. Qualitative findings included experiences in accessing care ranging from feelings of powerlessness to continuity of care challenges, influenced by geographical and financial barriers. In addition, experiences of patient-centered care encompassed empathetic practitioners, validation of concerns, and power dynamics, influenced by discrimination and advocacy for personalized support.

**Conclusions:**

This study offers an in depth understanding of experiences that have the potential to improve pregnancy care including targeted access to health services in remote areas, diminishing patient-provider hierarchies, culturally-sensitive care approaches for trust-building, and community engagement. Findings can inform healthcare professionals and policymakers about patients’ expectations of equity-oriented prenatal care.

## Introduction

Equity-oriented healthcare aims to improve health equity and patient experiences by tailoring care to the needs and priorities of individuals and populations, creating a safer and more accessible care environment [1]. While patients may have access to healthcare during pregnancy, the degree to which their care is equity-oriented can significantly impact their perceived satisfaction, decision-making around seeking care, and health outcomes for both them and their newborns [1]. Health inequities further shape care experiences, disproportionately affecting traditionally marginalized populations including individuals living with low income, those living in rural areas, and racial minorities, often perpetuating the inverse care law, where those most in need receive the least care [2]. These inequities, which are unjust and avoidable disparities in health and well-being [1], manifest in various ways through the social determinants of health, including language barriers that hinder health literacy, geographical isolation from specialized care, and financial constraints limiting access to essential nutrition and services [3,4].

Prenatal health is defined as overall maternal health during pregnancy [5]. Maternity care in Canada is primarily regulated by provincial and territorial health systems, with public health insurance covering most prenatal healthcare expenses, including routine screenings and consultations with healthcare professionals (HCPs). However, for some First Nations and Inuit populations, aspects of healthcare funding and governance involve federal jurisdiction [1,6]. Despite the availability of these services, prenatal care experiences remain deeply influenced by equity-oriented actions and health inequities [1]. For example, a qualitative study in Alberta found that immigrant women faced significant barriers to accessing and receiving quality care, including limited social support, cultural and linguistic challenges, and experiences of stereotyping and discrimination [6].

Although idealistically fair, Canada’s universal healthcare system may not always account for an individual’s social complexities. Some individuals may need more or different care (e.g., frequency of visits, specialized care) based on their social circumstances (e.g., low-income, newcomer status) and prior or current health (e.g., twins, gestational diabetes) [7]. The province of Ontario has the highest birth rate of 143,846 births per year from 2012 to 2024 accounting for approximately 40% of all births in Canada, [8] and the highest immigration retention rate of 93% reported in 2016, [9] compared to all other provinces and territories. Because of this diversity, Ontario may be a good representation of the populations across Canada [10]. Additionally, Ontarians who live in a rural region, are not born in Canada, or stay within the lowest income quintile, are at greater risk of maternal morbidity or mortality compared to those who live in urban regions, are born in Canada, or move up in income quintile, respectively [11–13].

To our knowledge, qualitative focused studies with a patient-centered emphasis on prenatal care to understand how health equity impacts care experiences are lacking across Canada and internationally [4].

This study aims to explore the care experiences of prenatal patients and examine the impact of health inequities on those experiences across Ontario. Specifically, it seeks to:

1. Explore patient experiences to inform future improvements for pregnancy care.
2. Identify how health inequities shape patient experiences and propose areas of focus to improve health equity.
3. Investigate patient perceptions and beliefs about how their experiences during pregnancy may impact the health and development of their newborn.

## Methods

### Study design

We conducted a patient-partner-oriented, cross-sectional, qualitative study from a pragmatist paradigm. A pragmatist worldview focuses on the problem to be investigated via the most appropriate methods and the subsequent consequences of the investigation [14–16].

#### Patient & partner engagement.

This work was conducted with team members who bring perspectives of pregnancy and pregnancy loss (author UKP) as a partner with lived or living experience (PWLE) and HCPs who have cared for prenatal patients (authors MHe and NI). These team members along with other partners (as outlined in S1 Table) were consulted at various stages of the study from design and recruitment to data collection and interpretation. We used the Guidance for Reporting Involvement of Patients and the Public Two (GRIPP2) checklist to ensure rigorous reporting of our patient and partner engagement processes (S2 File) [17]. PWLE were compensated for their contributions ($50 CDN per hour).

#### Conceptual framework.

This study was also guided by Cochrane’s PROGRESS-Plus health equity framework to ensure a comprehensive approach to consider issues that may lead to health inequity [3]. This framework was used as an explicit approach to implicitly integrate the social determinants of health into the study design as the framework is guided by the concept that health inequities are driven by systemic social factors. The PROGRESS-Plus framework categorizes factors of health equity into eight categories and leaves room for additional factors (Plus). Categories include: Place of residence (e.g., urban/rural), Race/ethnicity/culture/language, Occupation (e.g., unemployed), Gender/Sex, Religion, Education, Socioeconomic status (e.g., income), Social capital (e.g., marital status), and Plus (e.g., age) [3]. The PROGRESS-Plus framework was used to guide instrument development (surveys and interviews) and data analysis.

### Setting & participants

Participants eligible for this study included individuals 18 years and above, English speaking, lived in Ontario, who have been pregnant or experienced pregnancy loss (i.e., miscarriage, stillbirth, termination of pregnancy; at any gestational age) at any time within 12 months prior to recruitment, who have interacted with the healthcare system by either accessing or attempting to access any form of healthcare during their pregnancy (i.e., physical care, psychological or mental health care, virtual, in person, primary care, obstetrical care, midwifery, etc.) in the province of Ontario, Canada. Ontario was chosen as the sample site as it is the most diverse province in Canada, with the highest birth rate and immigrant retention rate [10].

### Sampling & recruitment

This study involved purposeful, maximum variation sampling to recruit participants with diverse prenatal experiences, focusing on those who may be at a disadvantage based on contextual experiences of inequity [18]. Across Ontario, recruitment efforts were supported by 1. Community Health Centres focused on individuals who have historically faced barriers in accessing health services, [19] 2. traditional organizations (e.g., The WOMB [World of My Baby]) and clinics (e.g., Ancestral Hands Midwives) focused on perinatal care, and 3. online parent Facebook groups. Recruitment started in May 2024 and ended in April 2025 and included strategies such as social media posts, e-newsletters, and printed posters which were either mailed out to specific sites or printed at the sites. Recruitment strategies specifically mentioned that the study was looking for participants whose experience had been impacted by “where they live, race and ethnicity, religion, gender identity, age, education, work, income, support system, or other circumstance” (S1 Fig). For a detailed list of recruitment partners and associated strategies, see S1 Table. Recruitment materials were reviewed by an individual with lived experience (UKP) and select recruitment partners (The WOMB and the Canadian Perinatal Mental Health Collaborative [CPMHC]) and adapted based on feedback.

Interested participants completed a short recruitment form to confirm their eligibility and to provide their contact information. The recruitment form was developed and administered online through Qualtrics (S3 File). Eligible participants were invited to complete a survey and participate in a virtual interview.

We aimed to recruit approximately 15–20 participants, and recruited until data saturation was reached when no new ideas related to the research objectives were identified [20]. This estimated sample size was chosen for reasons including the broadness of the research question, specificity of the type of participants, richness of conversations, and diversity of a maximum variation sampling technique [20]. No participants refused to participate or dropped out of the study.

### Data collection

Data collection instruments including a survey and interview were developed and piloted with a PWLE (UKP). To pilot the instruments, UKP completed the survey independently, and a mock interview was completed with UKP as the participant and ZL as the interviewer. To adjust the instruments based on feedback and user experience, UKP and ZL discussed the appropriateness of language used in questions, relevance of questions, and additional questions to consider [21]. Additionally, the survey and interview questions were sent to select partners (The WOMB, CPMHC, Country Roads Community Health Centre) for their asynchronous review, to ensure content was relevant to the study objectives; the partners largely agreed with the prepared instruments. Complete surveys and interviews can be viewed in the S4 and S5 Files.

#### Surveys.

Survey development was guided by the PROGRESS-Plus framework, was administered online through Qualtrics, and took approximately 5-minutes for participants to complete. The survey included questions about participants’ demographics (residence, employment, education, gender, race and ethnicity, religion, age) and their most recent pregnancy (high risk, first pregnancy, pregnancy outcome, and HCPs interacted with).

#### Interviews.

Interviews were guided by the PROGRESS-Plus framework and included questions about participants’ experiences of care during their most recent pregnancy, their expectations of HCPs, examples of how care met their needs, anything they would have changed during their care, how their care impacted their health and wellbeing and their newborn’s health and wellbeing, and how health equity factors may have played a role during their prenatal care. During interviews, participants were shown a list of the PROGRESS-Plus equity factors including age and disability and were asked to identify up to three factors, in no particular order, that they felt had the greatest impact on their care. Some selected fewer than three, while others identified more. The interviews were conducted virtually by ZL between October 2024 and April 2025, and were audio recorded using Zoom conferencing platform. Interviews were conducted in English and interview questions were also typed into the Zoom chat to enhance comprehension and accessibility. Field notes were taken during and after each interview. Interview transcripts were shared back with participants prior to commencing data analysis to give them an opportunity to correct or amend their transcript for accuracy. Each interview participant received an honorarium of $50 CDN for their participation. The interviewer did not have any prior relationships with participants.

### Data analysis & synthesis

Survey data was analyzed using descriptive statistics to describe participants’ demographic and pregnancy characteristics. Interview data where participants were asked to explicitly identify up to three PROGRESS-Plus factors that they felt impacted their care the most, was analyzed using descriptive statistics to explore trends of which equity factors impacted prenatal care experiences across participants. All descriptive statistics were completed using Microsoft Excel.

Interview audio recordings were transcribed, cleaned and de-identified by two researchers (MHo, ZL) in Microsoft Word. We applied a qualitative content analysis approach which involves the subjective interpretation and sense-making of data by a process of coding to identify patterns [22]. Interview transcripts were analyzed inductively using conventional content analysis to achieve study objective one by identifying findings related to patient experiences during pregnancy care, and objective three by identifying any perceived impacts on newborn health [22]. Transcripts were also analyzed deductively using directed content analysis following the PROGRESS-Plus framework to achieve study objective two by identifying findings related to how health equity impacts patient experiences [22]. A preliminary codebook was developed by ZL with insight from PWLE, UKP. The codebook was piloted with five researchers (ZL, MHo, TO, DT, RS) who inductively and deductively coded four transcripts independently, in duplicate, and then compared collectively to refine the codebook. Following the pilot coding process, the lead researcher (ZL) independently completed coding the remaining transcripts, with each coded transcript being reviewed by a second researcher (MHo, TO, DT, RS) for consistency. All qualitative analysis was completed using Microsoft Word. Study findings were discussed with PWLE, UKP, to ensure meaningful interpretation of the data.

A study highlights document was shared back with participants following analysis to provide participants with an opportunity to provide feedback on the relevance and appropriateness of findings. The highlights document included a brief summary of the study purpose, activities, and findings.

### Positionality statement

ZL, TO, and DT are PhD candidates at a Canadian university with expertise in health services and qualitative research. UKP is an individual with lived experience of pregnancy and pregnancy loss and a Registered Psychotherapist (Qualifying) and a Somatic Trauma Therapy Practitioner at a family wellness clinic. MHo is a recent BSc graduate from a Canadian University and a Research Assistant. ENA is a patient-engagement consultant and a person with lived experience in various areas of the health care system including fertility and pregnancy care. MHe is an Obstetrician and NI is a Family Physician, at a Canadian hospital. CL, OF, JS, NI, and RV are professors at Canadian Universities. As a team and as individuals, we reflected on our lived experiences and identities and discussed considerations of biases and values within this work.

### Ethical statement

This study has been approved by the University of Toronto Research Ethics Board (Protocol: 45685). All participants were given a study information sheet prior to engaging in data collection which included an overview of the study and dissemination processes for de-identified data. The survey included an implied consent statement prior to commencing survey questions and participants were required to complete an electronic written informed consent form via email prior to taking part in an interview. Consenting documents were collected by ZL. This work was guided by the COnsolidated criteria for REporting Qualitative research (COREQ) Checklist (S1 File).

## Results

### Participant characteristics

This study included 18 participants who completed a survey and semi-structured interview which ranged from 32 to 67 minutes. Participants’ self-reported sociodemographic and pregnancy characteristics are provided in detail in Table 1.

**Table 1:** Self-Reported Sociodemographic and Pregnancy Characteristics of Participants.

Characteristics (N = 18)	Count	Percentage
**Sociodemographic**		
**Region of residence in Ontario**		
Greater Toronto Area	13	72.2
Eastern	2	11.1
Northern	2	11.1
Central	1	5.6
**Citizenship Status**		
Canadian Citizen (Born in Canada)	11	61.1
Canadian Citizen (Not born in Canada)	2	11.1
Not a Canadian citizen or resident (e.g., Refugee, Temporary Citizen)	4	22.2
Permanent resident	1	5.6
Health Insurance*		
Ontario Health Insurance Plan (OHIP)	16	88.9
Health benefits/insurance through work	11	61.1
Private Insurance	3	16.7
No health insurance/coverage	1	5.6
Refugee claimants’ documents	1	5.6
**Employment**		
Full-time (currently or returning to full-time work)	11	61.1
Part-time (currently or returning to part-time work)	3	16.7
Self-employed	1	5.6
Unemployed – Looking for work	2	11.1
Unemployed – Unable to work for other reasons	1	5.6
**Highest Level of Education**		
Bachelors^*†*^	11	61.1
Diploma	2	11.1
Masters	2	11.1
Specialized Degree (e.g., medical doctor)	2	11.1
Highschool or equivalent	1	5.6
**Marital Status**		
Married, or in a domestic partnership	16	88.9
Single (never married)	2	11.1
**Individuals Living in Household**		
1	2	11.1
2	3	16.7
3	5	27.8
4+	7	38.9
Prefer not to answer	1	5.6
**Individuals Contributing to Household Income**		
1	4	22.2
2	12	66.7
3+	1	5.6
Prefer not to answer	1	5.6
**Household Income (CND)**		
Less than $50,000	2	11.1
$50,000 to $69,000	5	27.8
Above $70,000	10	55.6
Prefer not to answer	1	5.6
**Racialized Minority:** Yes	9	50.0
Ethnicity and Race*		
White (e.g., European descent)	5	27.8
South Asian (e.g., Indian, Pakistani, Sri Lankan)	4	22.2
Black (e.g., African, American, or Caribbean)	3	16.7
Other (e.g., Arab or Arab ancestry, East Asian, European/European ancestry, Indigenous, Latin or Hispanic ancestry, Persian, Southeast Asian)	7	38.9
Religion*		
Nothing in particular	7	38.9
Catholic	4	22.2
Christian	2	11.1
Agnostic	2	11.1
Other (e.g., Hindu, Mormon, Muslim, Orthodox, Protestant, Sikh)	3	16.7
**Age Group in Years**		
25-29	4	22.2
30-34	10	55.6
35-39	2	11.1
40 and over	2	11.1
**Most Recent Pregnancy**		
**First pregnancy:** Yes	10	55.6
**High risk pregnancy:** Yes	6	33.3
**Outcome of most recent pregnancy**		
Birth with no complications	9	50.0
Birth with non-life-threatening complications (e.g., premature, jaundice; but they are now healthy)	3	16.7
Pregnancy loss	2	11.1
Currently pregnant	4	22.2
Types of healthcare professionals interacted with*		
Primary care physician	16	88.9
Obstetrician/Gynecologist	9	50.0
Nurse	9	50.0
Midwife	8	44.4
Massage Therapist	7	38.9
Chiropractor	4	22.2
Pelvic Health Physiotherapist	3	16.7
Counsellor	3	16.7
Other (e.g., Acupuncturist, Osteopath, Reproductive Endocrinologist)	6	33.3

*Participants were allowed to select more than one option. †One participant did a graduate certificate following their Bachelors. CND: Canadian dollars.

All participants identified as being a woman and spoke English. The majority of participants were aged 30–34 years (n = 10, 56%). Most participants lived in the Greater Toronto Area in Southern Ontario (n = 13, 72%), while the others lived across Eastern (n = 2), Northern (n = 2), and Central Ontario (n = 1). The majority of participants were born in Canada (n = 11, 61%), covered by the provincial health insurance plan (n = 16, 89%), worked full-time and/or had an undergraduate bachelor’s degree (n = 11, 61%). Three participants were unemployed (17%) and two participants had a household income lower than $50K (11%). Half of participants identified as being a racial minority. (Table 1)

At the time of data collection, four (22%) participants were currently pregnant (one in their sixth month, and the other three in their eighth month), two (11%) had experienced a pregnancy loss, and 12 (67%) had delivered their baby, with majority of births having no complications. Over half of participants were experiencing their first pregnancy (n = 10, 56%) and a third reported being high-risk pregnancies (n = 6, 33%). All except two participants interacted with a primary care physician (n = 16, 89%), half of participants interacted with an OB and/or a nurse, and 44% (n = 8) interacted with a midwife (Table 1).

### Participant perspectives of pregnancy care & impact of health equity on experiences

**Fig 1:**
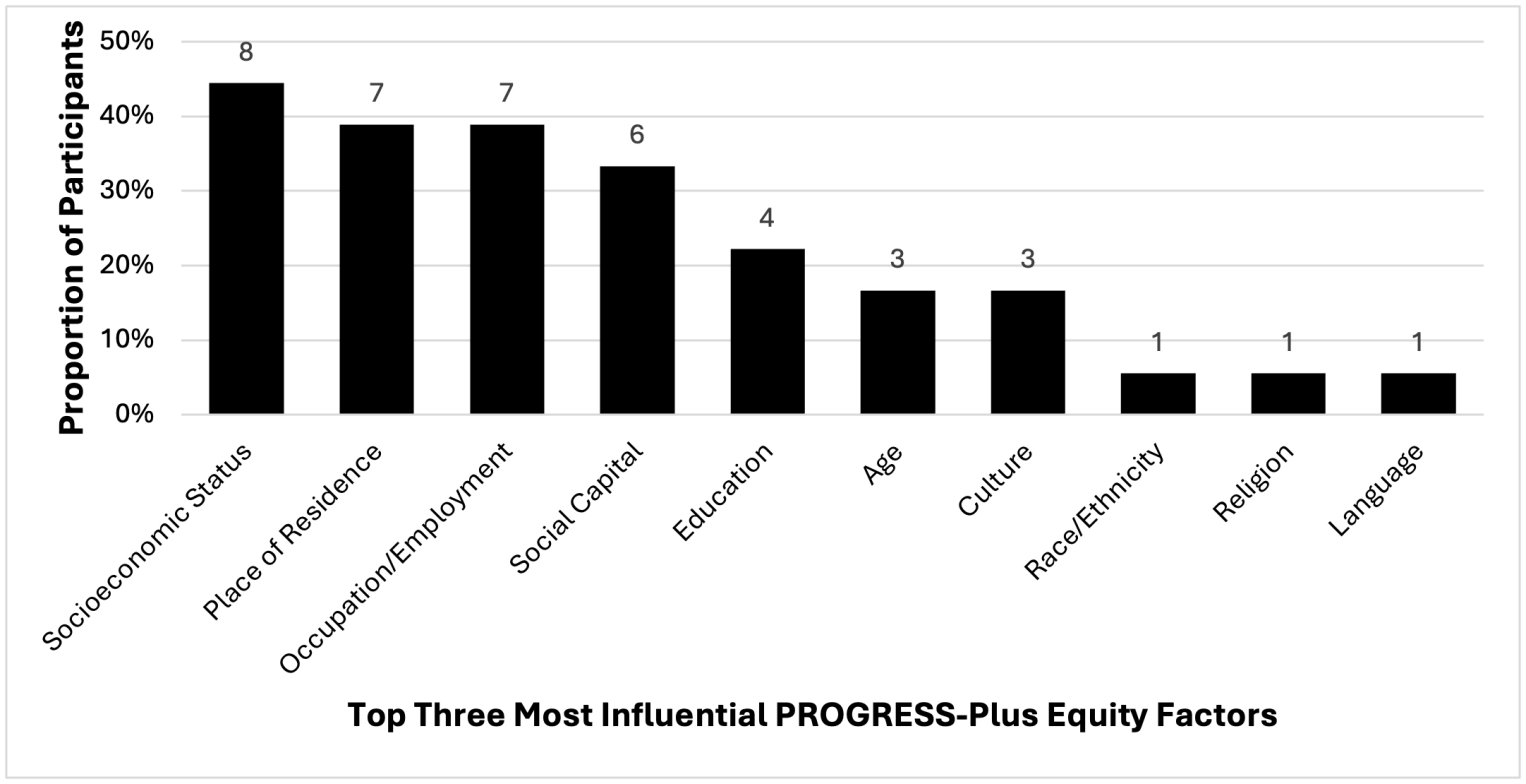
Proportion of PROGRESS-Plus equity factors reported by participants as being the top three factors that were most influential to their pregnancy experience.

We explored participant perspectives on their pregnancy care experiences. During interviews, participants were shown a list of the PROGRESS-Plus equity factors including age and disability and were asked to identify up to three factors, in no particular order, that they felt had the greatest impact on their care. Fig 1 illustrates the proportion of PROGRESS-Plus equity factors explicitly mentioned by participants when shown the list of the PROGRESS-Plus equity factors during interviews. Socioeconomic status was the most frequently cited factor, referenced by eight participants. Place of residence, and occupation and employment followed closely, with seven participants highlighting their impact. Gender, sex and disability were the only PROGRESS-Plus factors absent from participants’ top three most influential considerations.

Participant perspectives about their prenatal care experiences were organized into two categories: **Access to Care** and **Patient-Centred Approach to Care**. Participants’ perspectives on if their prenatal experiences had a perceived impact on the health and development of their newborn were integrated in these categories where applicable. These categories are described below and emphasized based on the findings in Fig 1 and participant demographics in Table 1. A list of additional example quotes from participants about the impact of health equity on their experience of pregnancy care are provided in S2 Table.

### Access to care

Participants’ experiences of accessing prenatal care varied widely, shaped by barriers such as geographic location, difficulties in finding a perinatal-specialized provider, continuity of care challenges, travel inconveniences, work obligations, and financial concerns.

Seven participants cited place of residence as a major factor affecting their care access (Fig 1). Rural participants faced long travel distances to specialized providers, requiring extensive planning and financial resources, adding layers of inconvenience to accessing care as described by a participant living in Northern Ontario:

*“[Distance to care] is a huge factor in terms of stress and planning. And you know, if things do go wrong throughout your pregnancy or there’s complications that come up, you’re not a 2-minute drive or even 20-minute drive from any sort of centre that can provide that extra care. You’re trying to either drive there in your own vehicle, 3 hours, take an ambulance, or getting flown out, and that alone just increases the stress of everything.” (P16)*

Securing a perinatal-specialized provider was difficult across both rural and urban settings, adding stress and uncertainty. Limited provider availability left some feeling vulnerable, forced to settle, and dependent on the healthcare system, as described by a participants’ challenge in finding an OB or midwife:

*“When I was applying for midwifery, I did get rejected 3 times from my zone, they were all full. So, I did additional research to see if I could find an OB and then the recommended OB rejected me as well because I wasn’t high risk. So, I was just finding it was a little bit difficult in the beginning to find the next step.” (P17)*

Participants valued consistency in their HCP from pregnancy through delivery and postpartum. Some benefited from this stability, while others struggled with fragmented care which was intensified for those with previous experiences of trauma. Although well-baby visits (i.e., routine check-ups with an HCP for infants and children) provided a means of access to care, participants expressed feelings of instability and unease when it came to finding care for their baby postpartum, particularly if they lacked a primary care professional. The challenge of care continuity is described by a participant who found it frustrating to repeatedly explain their medical history:

*“There was no consistency with who I saw. Like one day I could have seen a resident. The other time I could have seen my OB. The other time I could have seen another doctor. And it was always trying to keep up, to remind them of everything that had happened, even though it was on the chart. But like it gets to be so much information for them to review that I think sometimes it just gets lost.” (P12)*

Many struggled with taking time off work, whether due to concerns about diminishing their professional performance in the workplace, limitations in sick leave or vacation time, or the unfair reality of sacrificing income for essential medical appointments. Occupation and employment were reported explicitly by seven participants (Fig 1) and is described here by a participant that was concerned about taking time off work based on their younger age:

*“When you’re very young, you know it’s just very hard to take time off… and you have so much stuff that’s going on. You might have fears that, like your work performance is suffering. You might have fears that, like you’re gonna face repercussions.” (P06)*

Often at the expense of their earnings, participants had to navigate lengthy commutes, carefully coordinating time away from work, as reported by a participant who lives in the Greater Toronto Area, an urban region of Ontario, and earns a household income of $50-69K:

*“‘I’m doing my best, you know, subway is not working and I’m gonna try to get an Uber, just that I’m calculating, I might be slightly late’. [My HCP’s] assistant said ‘well, if you’re gonna be late, your appointment is gone, we’re gonna fill it by another patient, I’m sorry’, and I had to beg.” (P15)*

Financial challenges were reported by eight participants as a disadvantage based on socioeconomic status (Fig 1). Although essential and standard services during pregnancy are covered by the provincial health insurance plan in Ontario, limited insurance coverage (e.g., private or employer-based) heightened financial stress as participants grappled with affording critical services particularly for non-standard prenatal screenings and medications. Although not explicitly mentioned by participants, those with disabilities or co-morbidities reported requiring additional medical support, often leading to higher expenses that were not always manageable, as described by a participant who works part-time and earns a household income of $50-69K:

*“So, I wasn’t working, I was just on like a sickness [Employment Insurance]. So, I didn’t have a lot of income… But in the end, I did have to be put on insulin, and when they told me that, I was feeling really overwhelmed by that decision.” (P13)*

Notably, one participant acknowledged how their socioeconomic privilege including their racial identity and household income ($90K+), facilitated access to care they needed but made them ineligible for programs they wanted to engage in which were designed for marginalized groups. Others described how they actively overcame barriers to secure optimal care, citing the reassurance it brought them as described by a participant who works full-time with a household income over $90K, who chose to go out of their way for care:

*“We chose that hospital because it has the midwife unit in it. So, it’s a very specialized hospital. So, knowing that like, yeah, we’re going to have to drive far for it, but it sounds like it’s going to be worth it to us with the calm environment that it provides.” (P03)*

These findings illustrate the significant barriers participants navigated in accessing prenatal care, highlighting disparities rooted in geography, provider availability, and financial constraints.

### Patient-centered approach to care

Equity-oriented, patient-centered prenatal care varied widely, shaped by factors such as patient-provider trust, race and ethnicity, trauma-informed approaches to care, empathetic HCPs, advocacy for personalized support, and challenges navigating the health system.

Although explicitly mentioned by only one participant (Fig 1), race and ethnicity emerged as key influences on care experiences. Some participants described that they encountered bias from HCPs, leading to a loss of trust and reluctance to engage with the healthcare system. Feeling secure in their identity was critical for fostering meaningful patient-provider relationships. One participant of Southeast Asian ethnicity described their experience of discrimination which changed the trajectory of their care by having to choose a different hospital:

*“It was very, very upsetting and we felt really like unsafe at the hospital, like for him being Arab and for me being Filipino. We were just like, ‘this is not where we feel safe’.” (P14)*

While some participants sought accommodations such as being matched with a provider of the same race, culture, gender, or sex, workforce limitations often prevented this. However, HCPs’ ability to deliver empathetic and informed, person-centered care was seen as more important than the accommodations by participants:

*“It was something simple like she just put her hand on my knee and like, reassured me at that moment.” (P05)*

*“[Having an HCP of the same ethnicity] ended up not being that important in the end. I cared more about her experience at the end, that mattered more to me, her expertise.” (P14)*

Trust was a recurring theme, with participants wanting their concerns acknowledged, yet some felt dismissed:

*“[They] wrote [my health concerns] off as like, I didn’t know what I was talking about” (P04).*

Transparency and thoroughness in care conversations strengthened participants’ confidence in HCPs, while others described feeling powerless due to assumed treatment preferences and lack of informed consent as emphasized by this participant:

*“[The HCP] just went and did it [an episiotomy] and it wasn’t something that I had consented to at all. And this is where you know, again, I felt powerless. I felt like my choice was taken away from me in the sense and no one actually discussed it with me.” (P02)*

Newcomers to Canada often struggled with uncertainty about healthcare expectations. Some defaulted to passivity, fearing they lacked the right to request alternatives. Targeted supports such as translation services and newcomer networks helped some participants, and although only explicitly reported by three participants (Fig 1), culture as a barrier was prominent across others and is described in this participants’ prenatal experience in Canada as a permanent resident:

*“My lack of knowledge of the Canadian culture; it made me question my next move, I didn’t know what to do, didn’t know what was appropriate or was not.” (P15)*

Participants, especially first-time parents, valued being actively involved in care decisions beyond standard practices. Birth planning was valued by participants but often overlooked, leaving them uncertain and unprepared as described by this participant:

*“Even with like birthing plans or anything like that, you know, I was given a piece of paper and I had no idea what to do with it. [The HCP] would say, ‘write what you would like’, but I had no idea what to write because I didn’t know what to put in there.” (P12)*

Reported by seven participants (Fig 1), employment influenced care experiences, with some facing unsafe working conditions or long hours without accommodations as described by this participant in Northern Ontario who worked-full time:

*“I was working underground for the first four months of my pregnancy, and like I wasn’t feeling great support at work because I didn’t want to work underground.” (P08)*

HCPs often advocated on participants’ behalf, helping them secure adjusted workloads or leave options when possible. Conversely, socioeconomic status was reported by eight participants (Fig 1). Those living with low-income struggled to access necessities, finding the stress of choosing between economic stability and a safe pregnancy overwhelming. Participants often relied on financial assistance or donations as shared by an unemployed participant with a household income of less than 50K, who received financial support from their HCP because of their socioeconomic status:

*“[My HCP] do give us grocery cards and then they still give us food. Funny how they had many donations that come in if you want like, like I got diapers, wipes, I got clothes, I got a nursing bra, I got so many things from [the clinic] like they made the journey so easy.” (P10)*

Family, friends, and peers played a crucial role as was explicitly reported by six participants (Fig 1). Partners often served as primary advocates. While some HCPs actively involved partners in care decisions, this was not always accommodated. Partner involvement was highly valued as described by this participant:

*“[The HCP said], ‘come to whatever one you want to, you’re always welcome, it’s totally up to you.’ So it wasn’t like a shame thing either, but very much welcoming of like, [my partner] is welcome in this room just as much as I was.” (P03)*

Midwives or doulas were particularly valued for their advocacy during labor, believing their expertise was more effective than family members’ efforts, as described by this participant:

*“When you’re in the throws of labor, sometimes it’s hard to do [advocate for yourself]. So, I think it was just making sure that my advocate, so that would have been my husband but really more my doula - my doula knew exactly what I wanted.” (P02)*

Participants expressed a need for someone to rely on for emotional support, whether through personal networks or community-based support groups; some participants lacked this privilege, leaving them feeling isolated. Participants also believed that their emotional well-being and resilience could shape their baby’s future health and behavior, motivating them to actively seek resources and support to manage their mental health effectively. Past trauma and postpartum depression shaped participants’ care experiences; HCPs who took a trauma-informed, equity-driven approach were instrumental in fostering emotional safety and trust, as described by a participant:

*“I was feeling a lot of stress, a lot of pressure and like I was very anxious. And the nurse that I was seeing at the clinic, she was great. Like she would tell me ‘I will support you through whatever you need, if anything’s too much’, like she really cared about my stress and my mental well-being.” (P08)*

These findings highlight the importance of an equity-driven, patient-centered approach, demonstrating how trust, advocacy, and trauma-informed support shape prenatal care experiences.

## Discussion

Drawing on in-depth accounts from 18 participants in Ontario, this qualitative study explored how health equity factors influenced their experiences of prenatal care. The study sample was diverse and included individuals from urban and rural areas, those living with low-income, and identifying as a racial minority. Our findings highlight that socioeconomic status, place of residence, occupation and employment, and social capital are reportedly of importance for participants in accessing prenatal care and receiving patient-centered care. Culture, race, and ethnicity are also consistent as an underlying factor of inequitable experiences. Participants experienced challenges in accessing care including feeling powerless and a lack of care continuity. Patient-centred care experiences valued by participants included trauma-informed approaches to care, empathy, and advocacy, yet they were still faced by power-dynamics and discriminatory behaviours. As part of our third objective, we explored whether participants felt their pregnancy experiences influenced the health and development of their newborns. While these perspectives were not dominant in the data, recurring ideas emerged including how maternal stress and emotional wellbeing during pregnancy can impact a child’s future health and development.

A consistent theme across participants was access to care based on where they lived. Participants within Northern and Eastern Ontario expressed a lack of available services whereas those living in the inner-city of Toronto, Ontario expressed challenges in navigating public transportation. Our findings are consistent with the literature; a recent study by Orrantia and colleagues reported that the number of hospital sites not offering obstetrical care has risen in rural Northern Ontario by 37.5% and the number of primary care physicians that offer intrapartum care decreased by 65% in the last three decades [23]. Conversely, another study reported that although prenatal patients had access to care within an urban city in Manitoba, Canada, a lack of transportation was identified as a common barrier to accessing care for inner-city populations. Data from our findings and the literature speak to how healthcare interventions must be tailored to their surroundings, including place of residence along with other sociodemographic factors such as income [24].

Power dynamics between patient and provider were apparent across care experiences in this study. Participants expressed their disappointment in feeling powerless when it came to their health as they were often at the mercy of the HCP or the health system. Underlying causes for these power dynamics were believed to be related to culture, race or ethnicity, place of residence, and low-income, as reported by participants, which might have played a role in participants’ experiences alongside a lack of trauma-informed approaches to care. These suspicions are validated by other studies in the United States which have reported that patient-provider dynamics in pregnancy and birth were influenced by HCPs’ bias and judgement towards patients, and health care system structural issues [25]. Interestingly, some of our participants did not realize they were powerless or ‘settling’ until after they discussed their experiences in the interview. There is an opportunity for providers and patients to be collaborative in decision-making processes, lending to the idea of ‘power-with’ which is described as acting together for a collective outcome [26,27].

A meaningful finding of our results related to equity-oriented patient-centered care was the capacity of HCPs to accommodate patients’ needs in terms of culture, race, ethnicity, and/or gender or sex. For example, some participants explained how they would request (or wanted to request) a specific sex, gender, or race of their OB. The rationale for these accommodations is for patients to feel culturally safe and to build mutual trust with their provider [28]. However, the feasibility of this type of accommodation is low as Canada’s health workforce is already in a crisis and being able to find a ‘match’ to meet patient needs is often unrealistic [29]. Alternatively, regardless of HCP demographics, HCPs can be further trained and monitored for quality improvement of care based on patient-centered, equity-oriented, and trauma-informed approaches to care, so that their patients feel safe and satisfied [30]. This need to feel safe and satisfied was also cited by participants in our study and is consistent with a United States study that reported despite racial matching between patient and HCP, prenatal patients felt more comfortable with their HCP as they built trust over time [28]. This study also reported that they found no statistical difference between racial concordance and patient-centered care variables [28]. The idea of equity-oriented training for HCPs has been gaining interest over the last decade but has not been dominant in pregnancy care [31]. For example, continuing professional development policies in Canada only recommend trainings such as trauma- and violence-informed care, but do not mandate them for OBs [32]. Standardizing training would create a safer and more consistent environment for prenatal patients as they would have a reliable expectation across HCPs, regardless of practice location [31].

Interestingly, in our sample, those with low-income were provided with accommodations including donations and financial assistance and often expressed great appreciation and ease during their pregnancy journey; this is different from what is commonly reported in the literature. For inner-city residents living with low-income in the United States, the literature reports that they often experience challenges including food insecurity, paying for necessities, and lack of social supports during pregnancy [33,34]. It is important to note that majority of the low-income experiences within this study are from individuals that were attached to a primary care professional, often at a Community Health Centre. These centres focus on individuals who have historically faced barriers in accessing health services, [19] and are likely prepared with resources to accommodate low-income individuals. Therefore, we can infer that those living with low-income who are unattached or not connected to a Community Health Centre or similar organization, may not have the same experiences. Engagement between HCPs or HCP organizations and the community would facilitate ensuring financial needs and necessities can be met for low-income populations [35]. As reported in an international scoping review of community engagement in primary care by Erku et al., there is a potential role for community engagement initiatives to improve healthcare decision-making process and health outcomes [36]. If HCPs and healthcare organizations can create policies that outline how to meaningfully embed community engagement within their practice, to identify resources that are of need for their patient populations, then they would be more resource ready to care for marginalized populations.

### Limitations

There are limitations to report for this study. Firstly, our PWLE, UKP was instrumental in developing our recruitment strategy, yet it was still challenging to reach and recruit participants that were marginalized (e.g., those who were precariously housed, gender-diverse individuals, those who do not speak English). Our recruitment strategies included virtual connections through social media, email, and phone and could have been improved by actively engaging with communities in-person. Despite our comprehensive recruitment strategy, our study is limited to experiences of those who were connected to prenatal care as most of our recruitment strategies focused on existing prenatal networks, and therefore, we did not recruit anyone who did not have access to care during pregnancy. To increase equitable engagement in the study once recruited, in-person interviews and paper-based surveys were offered to potential participants, but these options were never used. Further, the population of focus within this study is already vulnerable and their pregnancy or postpartum challenges along with previous traumas and other life circumstances likely reduced peoples’ willingness to engage in the study as a participant. Second, Ontario is a large province with different communities in terms of rurality and culture and it is the most sociodemographic diverse province in Canada [10]. Because of this diversity, findings from this study can be generalized to other regions within and outside of Canada, while being mindful of specific contexts. There are vast differences in context even within Ontario which need to be taken into consideration when interpreting our findings. Although this data is not a complete representation of Canada, learnings can ignite discussions of how to improve equity-oriented prenatal care experiences across Canada and internationally. This study included people from across Ontario and attempted to generalize experiences while maintaining unique features of participants. Future studies can build on this work by focusing on engagement within specific communities or populations to further contextualize participants’ experiences and to create more targeted areas for future improvement. Thirdly, we used the PROGRESS-Plus framework to ensure we were comprehensive in our consideration of equity factors; however, the data collected in this study did not equally represent each of the equity factors as part of the framework, potentially limiting our comprehensive analysis. Lastly, this study only included patient perspectives; HCP perspectives are needed in future work to understand what is feasible to improve within prenatal care experiences.

## Conclusions

This study offers an in-depth understanding of care experiences across diverse individuals who have accessed prenatal care in Ontario, Canada. Our findings highlight actionable opportunities to improve pregnancy care experiences, including strengthening service delivery models to improve access to care in remote areas (e.g., expanding outreach services), addressing patient-provider power dynamics through communication and equity-oriented care training, and embedding culturally-sensitive approaches to build trust. The results also advocate for greater community engagement in prenatal program planning. Together, these findings can inform HCPs, health system planners, and policymakers in designing more equity-oriented prenatal care services.

## Supporting information

S1 File COREQ Checklist. (DOCX)

S2 File GRIPP2 Short Checklist. (DOCX)

S3 File Recruitment Form. (DOCX)

S4 File Survey Questions. (DOCX)

S5 File Interview Questions. (DOCX)

S1 Fig Recruitment Poster. (DOCX)

S1 Table Recruitment Partners and Strategies. (DOCX)

S2 Table Impact of health equity on participant experiences of pregnancy care, guided by PROGRESS-Plus framework. (DOCX)
