# Supplementary material for: Exploring prenatal care experiences in Ontario, Canada: An equity-oriented qualitative study
Source: PLoS One. 2026 Mar 30;21(3):e0345200. doi: 10.1371/journal.pone.0345200 (PMC13035144; doi:10.1371/journal.pone.0345200)
Supplement: S1 File — (DOCX) [file pone.0345200.s001.docx]

# S1 File: COREQ Checklist


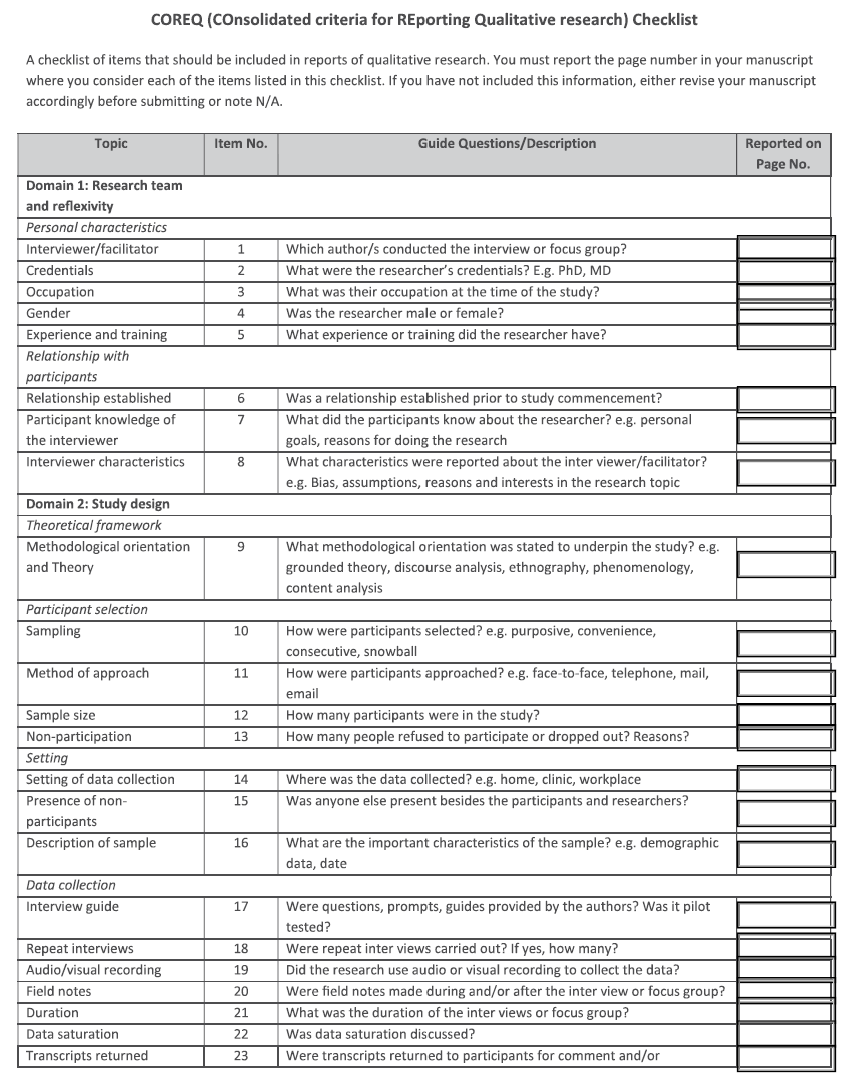


Data Collection

Positionality

Positionality

Not relevant

Positionality

Data Collection

Data Collection

Positionality

Study Design

Sampling & Recruitment

Sampling & Recruitment

Participant Characteristics

Sampling & Recruitment

Data Collection

N/A

Participant Characteristics

Data Collection

N/A

Data Collection

Data Collection

Participant Characteristics

Sampling & Recruitment

Data Collection


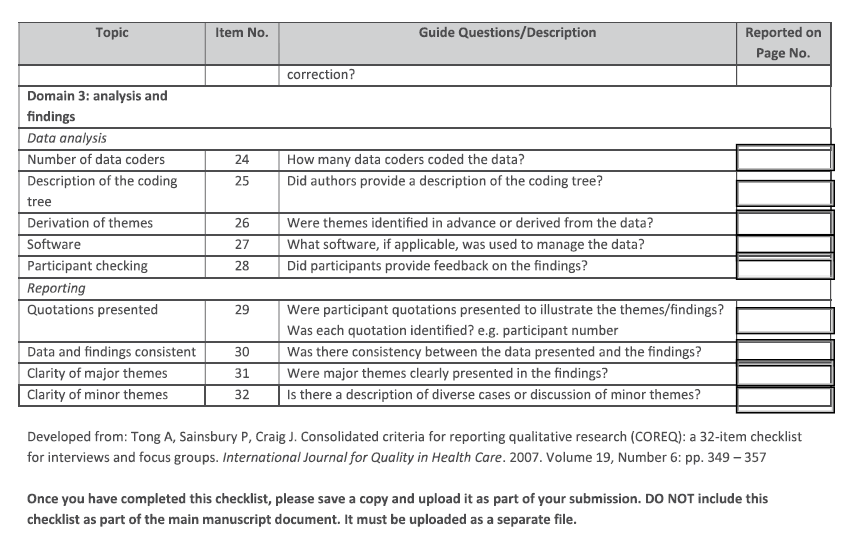


Data Analysis

N/A

Data Analysis

Data Analysis

Data Analysis

Results

Results

Results

Results
