# Supplementary material for: Exploring prenatal care experiences in Ontario, Canada: An equity-oriented qualitative study
Source: PLoS One. 2026 Mar 30;21(3):e0345200. doi: 10.1371/journal.pone.0345200 (PMC13035144; doi:10.1371/journal.pone.0345200)
Supplement: S2 File — (DOCX) [file pone.0345200.s002.docx]

# S2 File: GRIPP2 Short Checklist


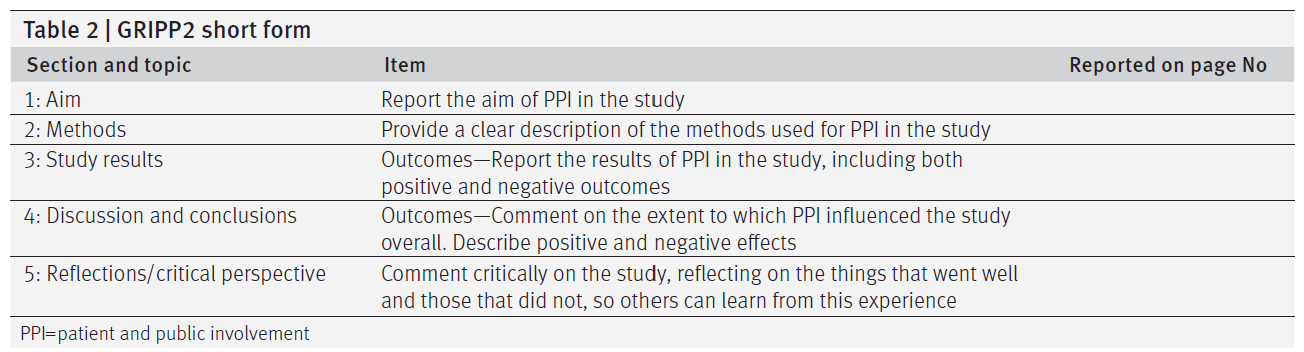


Introduction

Design, Data Collection & Analysis

N/A

Discussion

Limitations
