# Supplementary material for: Exploring prenatal care experiences in Ontario, Canada: An equity-oriented qualitative study
Source: PLoS One. 2026 Mar 30;21(3):e0345200. doi: 10.1371/journal.pone.0345200 (PMC13035144; doi:10.1371/journal.pone.0345200)
Supplement: S3 File — (DOCX) [file pone.0345200.s003.docx]

# S3 File: Recruitment Form

Thank you for your interest in participating in the study titled “Exploring equity in patient experiences of prenatal healthcare in Ontario”. To confirm your eligibility to participate in this study, please answer the following question:

1. Do all of the following criteria apply to you?
   - I am 18 years of age or older;
   - I have been pregnant within the last 12 months (including pregnancy loss);
   - I lived in Ontario while I was pregnant;
   - I accessed or attempted to access the healthcare system while I was pregnant.

- Yes, they all apply to me
- No

1. Thank you for completing this recruitment survey. Your eligibility will be manually confirmed by the research team. Please leave your email below and we will reach out to you with the more details regarding the actual study survey. [open text box for email]

Survey Complete

Thank you for your interest in participating in our study.
