# Supplementary material for: Exploring prenatal care experiences in Ontario, Canada: An equity-oriented qualitative study
Source: PLoS One. 2026 Mar 30;21(3):e0345200. doi: 10.1371/journal.pone.0345200 (PMC13035144; doi:10.1371/journal.pone.0345200)
Supplement: S4 File — (DOCX) [file pone.0345200.s004.docx]

# S4 File: Survey Questions

Section 1: Pregnancy Characteristics

1. *Please enter the dates of your most recent pregnancy (i.e., from when you found out you were pregnant to when you gave birth, the date you found out about your pregnancy loss, or your expected due date). (Month, Year to Month, Year; Example: May 2023 to September 2023) [open text box]
2. *Is this your first pregnancy?
   1. Yes
   2. No
3. *What was the outcome of your most recent pregnancy?
   1. Currently Pregnant – First Trimester (1-3 months)
   2. Currently Pregnant – Second Trimester (4-6 months)
   3. Currently Pregnant – Third Trimester (7-9+ months)
   4. Birth with no complications
   5. Birth with non-life-threatening complications (i.e., baby was born premature but is now healthy)
   6. Birth with life-threatening complications (i.e., baby was born with a neurodevelopmental disorder that last long-lasting effects on their lifestyle moving forward)
   7. Pregnancy Loss (i.e., miscarriage, stillbirth, ectopic)
   8. Option not listed – please specify [open text box]
4. *In your most recent pregnancy, were you told by your healthcare professional that you were a high-risk pregnancy?
   1. Yes
   2. No
   3. I’m not sure
5. *What types of healthcare professionals or other support providers did you interact with during your most recent pregnancy, prior to giving birth/pregnancy loss? [select all that apply]
   1. Family Doctor/General Practitioner
   2. Obstetrician/Gynecologist
   3. Midwife
   4. Nurse
   5. Doula
   6. Acupuncture
   7. Chiropractic
   8. Counselling
   9. Nutrition Support
   10. Naturopathic Medicine
   11. Traditional Healers
   12. Spiritual Support
   13. Massage Therapy
   14. Prenatal + Birthing Preparation Classes
   15. Pelvic Health Physiotherapist
   16. I did not interact with any healthcare professionals
   17. Option not listed – please specify [open text box]

Section 2: Demographics

1. *During your most recent pregnancy, where did you live? Please select a region and enter the name of the city in which you lived.
   1. Prefer not to answer
2. Central Ontario *(Muskoka-Kawarthas, Kitchener-Waterloo-Barrie and Hamilton-Niagara)* [open text box]
3. Eastern Ontario *(Ottawa and Kingston-Pembroke)* [open text box]
4. Greater Toronto Area *(Ajax, Aurora, Bradford West Gwillimbury, Brampton, Brock, Caledon, Clarington, East Gwillimbury, Georgina, Georgina Island, Halton Hills, King, Markham, Milton, Mississauga, Newmarket, Oakville, Oshawa, Pickering, Richmond Hill, Scugog, Toronto, Uxbridge, Vaughan, Whitby and Whitchurch-Stouffville)* [open text box]
5. Northern Ontario *(Northeast, Northwest, Thunder Bay and Sudbury)* [open text box]
6. Southwest Ontario *(London, Windsor-Sarnia and Stratford-Bruce)* [open text box]
   1. Option not listed – please specify [open text box]
7. *What is your current citizenship status?
   1. Prefer not to answer
   2. Canadian Citizen (Born in Canada)
   3. Canadian Citizen (Not born in Canada)
   4. Permanent Resident
   5. Not a Canadian Citizen or Resident
   6. Option not listed – please specify [open text box]
8. *What type of health insurance/coverage do you have? [select all that apply]
   1. Prefer not to answer
   2. Ontario Health Insurance Plan (OHIP)
   3. Private Insurance
   4. Health Benefits/Insurance through work
   5. No health insurance/coverage
   6. Option not listed – please specify [open text box]
9. *What is your current employment status?
   1. Prefer not to answer
   2. Full-time (Choose this if you are currently on maternity/paternity leave but will be returning to full-time work following your leave)
   3. Part-time (Choose this if you are currently on maternity/paternity leave but will be returning to part-time work following your leave)
   4. Casual/Contracted
   5. Self-employed
   6. Unemployed – Looking for work
   7. Unemployed – Unable to work for other reasons
   8. Option not listed – please specify [open text box]
10. *What is the highest level of education you have completed?
    1. Prefer not to answer
    2. Some of high school
    3. Highschool or equivalent
    4. Diploma
    5. Bachelors
    6. Masters
    7. Doctoral
    8. Specialized Degree (e.g., medical doctor)
    9. Option not listed – please specify [open text box]
11. *What is your current marital status?
    1. Single (never married)
    2. Married, or in a domestic partnership
    3. Widowed
    4. Divorced
    5. Separated
    6. Option not listed – please specify [open text box]
12. *How many individuals (including you) live in your household?
    1. Prefer not to answer
    2. 1
    3. 2
    4. 3
    5. 4+
13. *From the individuals that live in your household (including you), how many contribute to the total household income?
    1. Prefer not to answer
    2. 1
    3. 2
    4. 3
    5. 4+
14. *What is your approximate total household income level?
    1. Prefer not to answer
    2. Less than $50,000 CAD
    3. $50,000 to $69,000
    4. $70,000 to $89,000
    5. Above $90,000
15. *Gender identity: which of the following options best represent yours at this time? *[select all that apply]For definitions, please see Rainbow Health Ontario's glossary (*<https://www.rainbowhealthontario.ca/news-publications/glossary/>*)*
16. Prefer not to answer
17. Cisgender
18. Gender fluid
19. Man
20. Non-binary
21. Transgender
22. Two-spirit
23. Unsure/questioning
24. Woman
25. A gender identity we failed to list - please specify [open text box]
26. *Do you identify as a racialized minority and/or as belonging to a racialized community?

*Racialized, here, refers to individuals/groups who are attributed a racial/cultural identity based on their ethnicity/ancestry or skin colour. The term helps convey that ‘race’ does not have any genetic basis; rather it is a socially created category that has been used to sustain inequities.*

1. Prefer not to answer
2. Yes
3. No
4. Unsure
5. *Thinking about your ethnic/racialized/regional background or ancestry, do you identify with any of the following categories? [select all that apply]
6. Prefer not to answer
7. Arab or Arab descent (e.g., Egyptian, Syrian, Iraqi)
8. Black - African (e.g., Ghanaian, Kenyan, Somalian)
9. Black - North American or Caribbean (e.g., Barbadian, Jamaican)
10. Central Asian (e.g., Kazakh, Afghan, Uzbek)
11. East Asian (e.g., Chinese, Japanese, Korean)
12. European/European ancestry
13. Indigenous (e.g., First Nations, Inuit, Métis)
14. Jewish (e.g., Sephardic, Ashkenazi, Maghrebi, Ethiopian)
15. Latin or Hispanic descent (e.g., Argentinian, Chilean)
16. Mixed heritage
17. Persian (e.g., Iranian, Afghan, Tajik)
18. South Asian (e.g., Indian, Pakistani, Sri Lankan)
19. Southeast Asian (e.g., Cambodian, Laotian, Thai)
20. Western Asian (e.g., Lebanese, Kuwaiti)
21. White (e.g., European descent)
22. A group we failed to list - please specify [open text box]
23. *Are you a member of a religious group? [select all that apply]
    1. Prefer not to answer
    2. Buddhist
    3. Catholic
    4. Hindu
    5. Jewish
    6. Mormon
    7. Muslim
    8. Orthodox
    9. Protestant
    10. Roman
    11. Atheist
    12. Agnostic
    13. Nothing in particular
    14. A group we failed to list - please specify [open text box]
24. *What age group do you identify with?
    1. Prefer not to answer
    2. 18-24 years
    3. 25-29 years
    4. 30-34 years
    5. 35-39 years
    6. 40-44 years
    7. 45-49 years
    8. 50 years and over
25. *Do you feel confident in speaking, reading or writing in the following languages? [select all that apply] [Matrix question]
    1. English: Prefer not to answer, speaking, reading, writing, none or not applicable
    2. French: Prefer not to answer, speaking, reading, writing, none or not applicable

Survey Complete

Thank you for participating in our study.
