# Supplementary material for: Exploring prenatal care experiences in Ontario, Canada: An equity-oriented qualitative study
Source: PLoS One. 2026 Mar 30;21(3):e0345200. doi: 10.1371/journal.pone.0345200 (PMC13035144; doi:10.1371/journal.pone.0345200)
Supplement: S5 File — (DOCX) [file pone.0345200.s005.docx]

# S5 File: Interview Questions

1. How did you find out you were pregnant and what did you do next for your care?
2. In your first trimester, did your healthcare provider talk to you about prenatal screening options? **(If no, skip to question #3)**
   1. How did your healthcare provider bring up the topic of prenatal screening? Can you tell me about the conversation?
   2. Was there anything that was helpful or supportive in you being able to take the prenatal tests that were offered to you? Anything that was a barrier?
3. If you were informed about prenatal screening, do you think this would have changed the trajectory of your care?
4. Now thinking about your overall pregnancy journey, of all the healthcare providers you interacted with, which one would you say had the greatest impact on your care, prior to giving birth/pregnancy loss, and why?
   1. Is that different from the provider you interacted with the most?
5. What are some ideal characteristics, qualities or practices of healthcare professionals or other support providers that you expect when seeking care during your pregnancy, to ensure the care you receive is fair?
6. Can you provide an example of a time when a healthcare provider equitably met your needs during pregnancy?
7. Can you provide an example of a time when a healthcare provider did not meet your needs during pregnancy?
8. Is there anything you would change about the care you received during pregnancy, to make the experience fit your needs better?
9. Do you feel that your journey and experiences of care during your pregnancy would/will influence your health and wellbeing during or after pregnancy?
   1. Do you feel that it would/will influence the health and wellbeing of your newborn?
10. Looking at this list of factors that might have shown up as barriers to your care during pregnancy, can you choose the top 3 that were most influential, and explain why. The top 3 don’t need to be chosen in any specific order.
    1. List of Equity/Inequity Factors

- Location of residence or distance to care
- Race
- Ethnicity
- Language
- Culture
- Religion
- Occupation or employment
- Gender identity or sex
- Education level
- Socioeconomic status or income level
- Social capital or support system
- Age
- Disability (physical)
- Disability (non-physical)
- Other

1. Is there anything else you would like to share about your pregnancy experience that we haven’t yet talked about?
