# Supplementary figures and images for: Exploring prenatal care experiences in Ontario, Canada: An equity-oriented qualitative study
Source: PLoS One. 2026 Mar 30;21(3):e0345200. doi: 10.1371/journal.pone.0345200 (PMC13035144; doi:10.1371/journal.pone.0345200)

**S1 Fig. Recruitment poster printed at sites, used in e-newsletters, and in social media posts.**


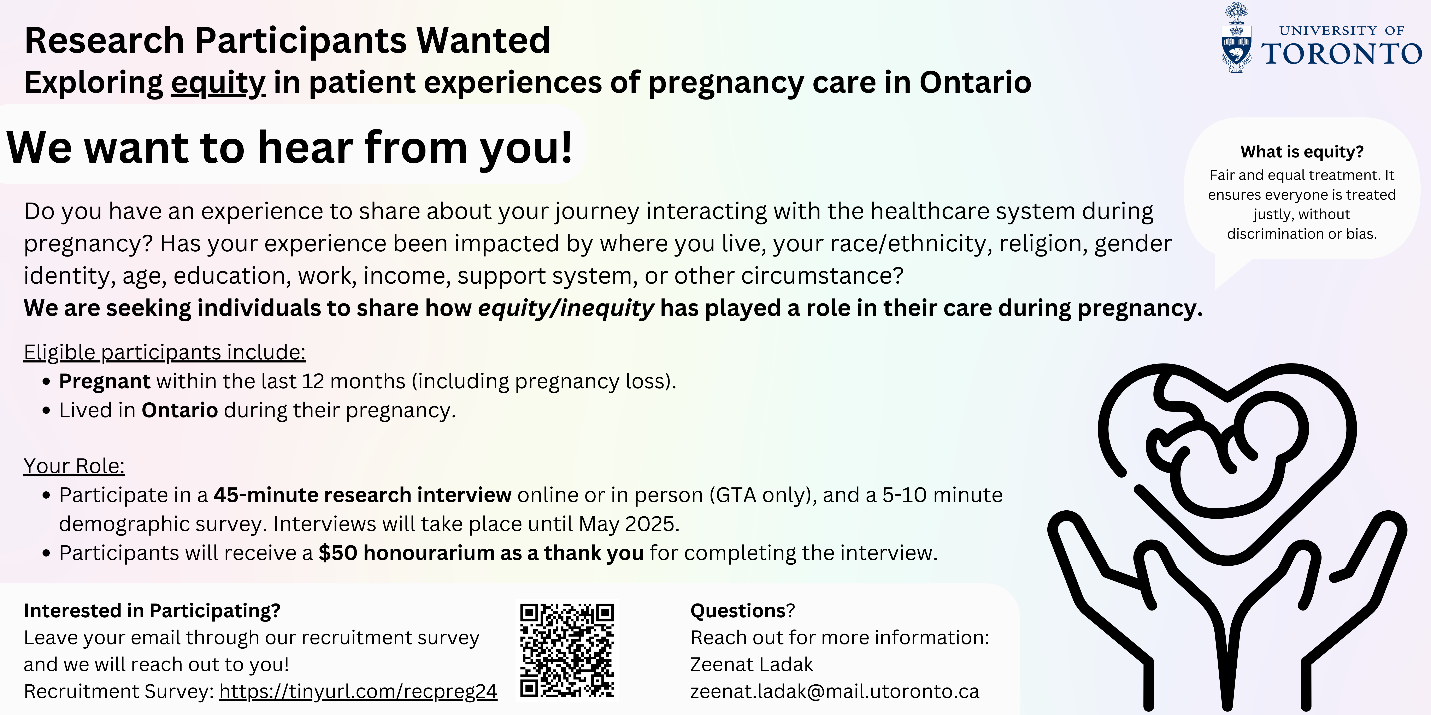

Supplement: S1 Fig — (DOCX) [file pone.0345200.s006.docx]
