# Supplementary material for: Exploring prenatal care experiences in Ontario, Canada: An equity-oriented qualitative study
Source: PLoS One. 2026 Mar 30;21(3):e0345200. doi: 10.1371/journal.pone.0345200 (PMC13035144; doi:10.1371/journal.pone.0345200)
Supplement: S1 Table — (DOCX) [file pone.0345200.s007.docx]

# S1 Table. Recruitment partners and strategies.

| Social Media Posts |
| --- |
| - Rainbow Health Ontario - Midwives of Lindsay - “Ukranian Moms in Toronto” Facebook Group - “Ukranian Moms+Dads in Toronto and the GTA” Facebook Group - “Toronto Moms” Facebook Group |
| E-Newsletters |
| - Canadian Perinatal Mental Health Collaborative - Department of Applied Psychology & Human Development at the Ontario Institute for Studies in Education |
| Printed Posters |
| - The WOMB (World of My Baby) - Kingston Community Health Centre - Country Roads Community Health Centre - Midwives of Lindsay - Ancestral Hands Midwives - Parkdale Queen West Community Health Centre - CommunitiCare Health, Four Villages Community Health Centre - Marathon Family Health Team (Dr. Elias Orrantia) |
