# Supplementary material for: Exploring prenatal care experiences in Ontario, Canada: An equity-oriented qualitative study
Source: PLoS One. 2026 Mar 30;21(3):e0345200. doi: 10.1371/journal.pone.0345200 (PMC13035144; doi:10.1371/journal.pone.0345200)
Supplement: S2 Table — (DOCX) [file pone.0345200.s008.docx]

# S2 Table. Impact of health equity on participant experiences of pregnancy care, guided by PROGRESS-Plus framework

| **Example Quotes** |
| --- |
| **Place of Residence** |
| *“That wasn't an option here, so we had to go to Thunder Bay.”(P08)* |
| *“I live far from my work too. It's about an hour commute. It's hard to make an appointment before your shift starts because you still have to drive an hour to work and vice versa. So sometimes it does limit the accessibility to have appointments unless I take the full day off.”(P03)* |
| *“If you can drive and you're young like you're ‘OK’. And then if you don't, then you're kind of pooch… I'm very much aware if I didn't have a license, I would not have access to healthcare here or my husband would have to take more time off work and not get paid so he could drive me and that would affect our income.” (P03)* |
| *“I was grateful, but because of the whole the whole distance, you know from downtown Toronto to Thornhill… I wanted to change family doctors, and I wasn't liking the experience anymore.” (P15)* |
| *“With the shared care, it allowed me to stay in [my town] for the majority of my prenatal appointments. It meant not having to take time off work, scheduling it around. You know my son being in daycare here, my husband travels for work a lot, so also trying to navigate around that and being home. We did some phone appointments in place of in person appointments.” (P16)* |
| **Race/Ethnicity/Culture/Language & Religion** |
| *“We speculated about whether my race had something to do with it, with the way I was treated with my first [baby]. But there was nothing overt about it, it was just a feeling. My husband is Caucasian, so we have two mixed raised babies. And this is where I don't want to accuse anyone of being racist, right? But  I often wonder if that influences the way we were being treated.” (P02)* |
| *“Just somebody who could relate to me since I'm Filipino, who was, like, compassionate… and that I wouldn’t experience discrimination.” (P14)* |
| *“Maybe the culture is another thing because we are new here. We do not know how some things have been done here.” (P10)* |
| *“So, [my HCP] would explain that, ‘oh, that is not a standard in Canada, here's how things go’, so she did give me more time during my appointments, which she did explain how things work more thoroughly.” (P15)* |
| *“Where I'm from, whatever you get what you get. It’s just the way it is. So, you don't get to ask for anything else.”* |
| *“I think that like being able to speak fluently in English and things like that like directly impacted the services that I was getting, I can probably imagine that could have been a very strong barrier to receiving care here.” (P07)* |
| *“Just tough to find people that like are in your demographic for the support you want. It's hard. It's not the easiest thing. I think especially in my location. So that's why I had to go elsewhere to find what I was looking for.” (P17)* |
| *“I'm African, I'm Black, I'm Nigerian. So sometimes when I go to some specific places, you know, there are some things people don't need to say to you, you can just read between the line…* *I'm proud to be black, and it's not a crime to be black.” (P18)* |
| **Occupation & Socioeconomic Status** |
| *“It was a new job, and I was new in the country, so it was a very big deal for me to not lose my job, to not have problems. And so, I really, really appreciated that [the doctor] did that and she wanted to make sure that I still got my appointment, I got my ultrasound. And all of that it really meant a lot.” (P15)* |
| *“I do find the like amount of appointments and stuff is a little overwhelming, and with my job it's hard to sometimes… it's a lot of like juggling to get the days off to go. I obviously could use a sick day if I had to, but I try to avoid that.”  (P03)* |
| *“Because of like my complications and things like that, [the HCP] did try to push for accommodations for work and things like that, and like informing my workplace, like what the challenges were and helping me support in that sense with accommodations at work.”(P07)* |
| *“I just couldn't afford it and I know that that was because of my income level, it's also ridiculously expensive to live here in this province. Rent is insane. So, I yeah, it just feels like a lot of our money goes to just like basics and I don't have any insurance or anything. So yeah, it feels like a lot of my care... People would recommend things and then just realize that I couldn't afford it, and so then it wasn't available to me or a couple of times, I had to choose to do it anyways.* *I give up a lot because of our income level.” (P13)* |
| *“It's obviously not easy to travel three hours and like, find accommodations and pay for all of that, like all the expenses, gas, hotel, food, is all very expensive. So yeah, that made a big impact on my experience.” (P08)* |
| *“It's not necessarily fair that you do have to pay for [prenatal screening]… for example like it wasn't too expensive, but like obviously it is still expensive, in the sense that, like you do kind of feel bad when you're paying for it, you're like, oh, I do have to spend money on this versus like something that should be easily accessible for people. So, I did kind of feel like I have to pay for this and like, what if the results don't even show anything?... also when they explain the results and they tell you that for example, there could be a risk of like false negatives, you're kind of like, OK, I'm essentially might be spending money, but it might just be for nothing.” (P06)* |
| *“I wanted to take mental health days and just time off, and I didn't have much of that. So, there was a time where I requested [unpaid time off] work and they declined it. And that really made me like, feel stressed out.” (P17)* |
| **Gender or Sex** |
| *“I actually wanted like a female, Filipino health care provider specifically, so I was kind of selective. And I got lucky and that my primary care doctor was Filipino and female. And then she referred me to an OB that was coincidentally also female, Filipino.” (P14)* |
| *“When it came to my options there she really explained like every single option, she even related them to her as a woman and she told me her own experiences… So, I had a very good idea of what I wanted.” (P15)* |
| **Education** |
| *“If they know you're a healthcare professional, they talk to you in a in a different way, maybe use more scientific terminology that makes it like, gives you more information, because maybe you'll understand it more.” (P05)* |
| *“But I do think that I have a bit of a privilege on that sense because I do work at our family health team. I have experience in research in [OB]. So, I think that just the world that I work in has given me an advantage in that sense… just to know my options and know what's available.” (P16)* |
| **Social Capital** |
| *“I found that to be really helpful and just taking the time to make it more personal by like leaving us with pictures or bringing in my partner to let him see and explaining certain things.” (P07)* |
| *“Because of the timing of the high-risk clinic I was never able to have my [my partner] with me at appointments which was a challenge for me with my emotional health. And so, at that point, I was just so overwhelmed they had all these people coming in and telling me all of these things.” (P13)* |
| *“So I did have some people that recently had babies or were pregnant the same time as me. It was nice to have them to talk about how I was feeling and like what I was going through. So, I felt like pretty supported, I was fortunate for that.” (P08)* |
| *“So where I volunteered there, we had some friends, very nice people… They are the first family we have here in Canada, those people were people that were with me during the pregnancy; my daughter was [with them] because she could not go to the hospital with us. She I spent like 2 days there, I was like, ‘oh, ok, and she was good because they are family already.” (P10)* |
| *“I think that kind of shed light on some like, Wow, this is systemic and like I'm glad I'm getting a labour doula to help advocate for me if she does see racism.” (P14)* |
| *“I have my mom or my sibling, but I do not have the resources to bring them over. It's a lot. Sometimes I get so angry because right now I'm home alone… I wish there is a friend or family that could help me with this, so then it occurred to me like I'm only alone in this. I know my partner is there, my husband, but he's managing to do like a small job by the side or part-time job, which Is barely enough for what we have, but I just have to do like this.” (P18)* |
| **Plus: Age** |
| *“They really treat you differently based on their clinical guidelines once you cross over the age of 40… I guess I was concerned about my age.” (P02)* |
| *“I find that because of my age and the fact that I was a first-time mom, I wasn't listened to as well as I would have been had I have already had the experience of like being a mom prior to this pregnancy.” (P04)* |
| **Plus: Disability & Co-Morbidity** |
| *“Both physical and non-physical 'cause I have a disability that people may not see as a disability… I think a lot of doctors are very easy to dismiss those things and say that I don't have it or that it doesn't impact, which truly it does.” (P09)* |
| *“I had to be put on temporary disability this pregnancy, I wasn't able to work because of a lot of issues with my hips and like sciatica.” (P13)* |
